# Lessons learned from implementing a diversity, equity, and inclusion curriculum for health research professionals at a large academic research institution

**DOI:** 10.1017/cts.2024.6

**Published:** 2024-01-12

**Authors:** LaMisha Hill Weller, Anna D. Rubinsky, Starley B. Shade, Felix Liu, Iona Cheng, Georgina Lopez, Asha Robertson, Jennifer Smith, Kristina Dang, Christian Leiva, Susan Rubin, Suzanna M. Martinez, Kirsten Bibbins-Domingo, Meghan D. Morris

**Affiliations:** 1 Department of Obstetrics, Gynecology and Reproductive Sciences, University of California, San Francisco, CA, USA; 2 Office of Diversity and Outreach, University of California, San Francisco, CA, USA; 3 Department of Epidemiology and Biostatistics, University of California, San Francisco, CA, USA; 4 Department of Medicine, University of California, San Francisco, CA, USA

**Keywords:** Academic research institution, diversity training, public health, anti-racism

## Abstract

**Objective:**

Despite advances in incorporating diversity and structural competency into medical education curriculum, there is limited curriculum for public health research professionals. We developed and implemented a four-part diversity, equity, and inclusion (DEI) training series tailored for academic health research professionals to increase foundational knowledge of core diversity concepts and improve skills.

**Methods:**

We analyzed close- and open-ended attendee survey data to evaluate within- and between-session changes in DEI knowledge and perceived skills.

**Results:**

Over the four sessions, workshop attendance ranged from 45 to 82 attendees from our 250-person academic department and represented a mix of staff (64%), faculty (25%), and trainees (11%). Most identified as female (74%), 28% as a member of an underrepresented racial and ethnic minority (URM) group, and 17% as LGBTQI. During all four sessions, attendees increased their level of DEI knowledge, and within sessions two through four, attendees’ perception of DEI skills increased. We observed increased situational DEI awareness as higher proportions of attendees noted disparities in mentoring and opportunities for advancement/promotion. An increase in a perceived lack of DEI in the workplace as a problem was observed; but only statistically significant among URM attendees.

**Discussion:**

Developing applied curricula yielded measurable improvements in knowledge and skills for a diverse health research department of faculty, staff, and students. Nesting this training within a more extensive program of departmental activities to improve climate and address systematic exclusion likely contributed to the series’ success. Additional research is underway to understand the series’ longer-term impact on applying skills for behavior change.

## Introduction

Diversity, equity, and inclusion (DEI) are imperative in supporting intersections of health science education, public health, and research. It is well documented that diverse teams outperform homogenous teams in business settings, however, these findings also extend to healthcare settings [1,2]. Authors Gomez and Bernet summarized diversity research specific to healthcare to better understand the relationship between diversity, innovation, patient health outcomes and financial performance. Their meta-analysis affirms that diverse teams are related to improved patient care. In addition, the authors found that diverse healthcare teams demonstrate increased innovation, communication, and risk assessment. Finally, the authors confirmed that diversity-friendly environments support change management. Within healthcare these domains are central to health equity and workplace belonging [3]. The fields of epidemiology and public health have made significant contributions to best practices in defining and using race and ethnicity in research; however, many opportunity gaps remain surrounding diversity, equity, and inclusion in the field [4]. Following the COVID-19 pandemic [5], the murder of George Floyd [6], and a rise in anti-Asian hate [7] there was a recommitment across biomedical and health professional institutions to develop diversity initiatives to address anti-racism, diversity education and curriculum, and equity in recruitment and retention [8].

In 2020, the Public Health National Center for Innovations launched a task force to review the 10 Essential Public Health Services (EPHS) centered on equity and removal of systemic and structural barriers (e.g., poverty, racism, gender discrimination, ableism, and other forms of oppression) that result in health inequities [9]. The eighth EPHS mission was to “Build and support a diverse and skilled public health workforce.” Potential steps to address diversity and inclusion in public health include culturally congruent education and training for professionals and leadership and nurturing diverse future public health practitioners. The public health and health sciences field, however, lacks diverse racial and ethnic representation and remains overwhelmingly White [10–12]. There have been efforts to incorporate diversity and structural competency, defined as the ability to recognize and intervene on structural factors that impact health inequalities, as core tenants in medical education, but these teachings are still needed for those in public health research [13,14].

Diversity training can be defined as a distinct set of instructional programs to facilitate positive intergroup interactions, reduce prejudice and discrimination, and enhance skills, knowledge, and motivation to interact with diverse individuals [15]. There are several core aspects of successful diversity training. A meta-analysis of 260 independent studies found greater positive effects when diversity training was targeted to awareness and skills development, conducted over time, and utilized a comprehensive approach [16]. Rooted in organizational development theory, diversity training sessions that utilize a comprehensive approach to identifying and intervening on structural factors are more efficacious than trainings that focus primarily on individual attitudes. A comprehensive diversity training includes support from senior leadership, is tailored to the specific organization, connected to the group’s operating goals, inclusive of employees from all levels, and facilitated by content experts [17,18].

We conducted a literature review within the Journal of Clinical and Translational Science and using the keywords “diversity, equity, inclusion, and curriculum.” The results identified 6 relevant articles that focused on curriculum development for trainees [19,20], utilization of diversity committees [21,22], and faculty development [23,24]. Enders and colleagues evaluated faculty and staff attitudes to a Health Science Research (HSR) departmental Diversity, Equity, and Inclusion (DEI) plan. Authors found that many respondents expressed support for the proposed initiatives of a blinded promotion review process and DEI training. Interestingly, authors observed that attitudes toward the proposed initiatives differed by faculty and staff subgroups. Specifically, more faculty and staff from racial and ethnic minority groups supported inclusion nudges and blinded promotion reviews compared to White and non-Latinx individuals [25]. Our curriculum furthers the existing body of literature by leveraging our departmental diversity committee to inform our workshop curriculum for a health science academic department that is inclusive of faculty and non-faculty researchers.

We developed and implemented a four-part DEI curriculum for health research faculty, staff, and researchers at an academic health science institution. Four 2-hour workshops combined didactic and experiential activities rooted in transformative learning and critical race theories [26–28]. Transformative learning theory suggests that paradigm shifts occur through critical self-reflection, acquiring new knowledge, building competencies, and integrating new schemas [28,29]. Critical race theory is grounded in four pillars including race consciousness, contemporary orientation, centering in the margins rather than the mainstream, and theory-informed actions, also known as praxis [27]. Combined, they provide an excellent framework to support a curriculum that provides evidence-based diversity content and reveals the often invisible forms of inequity and tools to support equity and allyship at individual and structural levels.

The workshop series was designed to increase foundational knowledge of core diversity concepts and improve skills to support an inclusive population health science academic department. We describe the curriculum development and implementation and evaluate the program’s efficacy.

## Methods

### Setting

The 250-person Department of Epidemiology and Biostatistics was comprised of 51% faculty, 41% staff (including non-faculty academics), and 8% trainees (doctoral students or postdoctoral trainees). Self-reported race and ethnicity was 58% White, 27% Asian and Asian American, 6% Black and African American, and 5% Hispanic/Latino(a); more staff than faculty identified as a member of an underrepresented racial and ethnic minority (URM) group (27% vs. 7%, *p* < 0.0005). Sixty-two percent were women.

### Workshop development and curriculum content

The DEI committee, consisting of 14 department members (6 faculty, 7 staff, and 1 student), developed the four-part DEI curriculum for a 250-person department of epidemiology and biostatistics. The committee partnered with a campus DEI professional (LHW) to develop the curriculum and serve as the lead facilitator. She developed all workshop content using feedback from an all-departmental needs assessment survey and conversations with committee members. Committee members assisted with workshop logistics and facilitated small group activities. The goal was to increase overall understanding of core DEI concepts and skills for inclusive behaviors. Our methodological framework was based on the Six-Step Approach to curriculum development [30].

### Step 1: Problem identification and general needs assessment

Current scholarly work on diversity training and workshops in public health and academic health science institutions reveals underutilization of a multisession approach. In addition, extant literature mainly focused on education or didactic information, leaving out opportunities to examine experiential and skill-based curriculums [31,32].

### Step 2: Targeted needs assessment

The month before developing the workshop curriculum, we administered a short survey to evaluate the level of DEI knowledge and attitudes and to assess the department members’ content preference. Of the 100 respondents, 50% were staff, 34% faculty, and 16% trainees. Most (71%) had been in the department for less than 5 years and 62% did not have prior DEI training. Among those with previous training (*n* = 34), participation was based on personal interest (65%), a requirement (44%), or professional development (47%). Supplemental Table 1 describes respondents’ preference for workshop session topics and perceived disparities across workplace areas. Information and feedback from the needs assessment survey informed the workshop series topics and curriculum content.

**Table 1.** Presession participant characteristics, overall and by department of epidemiology and biostatistics diversity, equity and inclusion workshops, 2019–2020

		Workshop 1	Session 2	Session 3	Session 4	
	Overall	*N* = 67 Freq (%)	*N* = 54	*N* = 45	*N* = 82	*p*-value*
Role						0.070
Student	(5)	4 (6)	1 (2)	3 (7%)	5 (6%)	
Post doc	(6%)	5 (7)	0 (0%)	2 (4%)	7 (9%)	
Staff	(58%)	28 (42%)	38 (70%)	29 (64%)	48 (59%)	
Non-faculty academic	(6%)	6 (9%)	1 (2%)	3 (7%)	5 (6%)	
Faculty	(25%)	24 (36%)	14 (26%)	8 (18%)	17 (21%)	
Hire/supervise personnel						0.48
Yes	(43%)	31 (46%)	21 (39%)	15 (35%)	39 (48%)	
No	(57%)	36 (54%)	33 (61%)	28 (65%)	43 (52%)	
Years worked in department						0.24
Less than 5	(71%)	49 (73%)	28 (56%)	29 (74%)	61 (77%)	
5–10	(9%)	5 (7%)	8 (16%)	2 (5%)	6 (8%)	
More than 10	(20%)	13 (19%)	14 (28%)	8 (21%)	12 (15%)	
Gender						0.26
Male	(25%)	18 (27%)	17 (31%)	12 (27%)	14 (17%)	
Female	(74%)	49 (73%)	37 (69%)	31 (70%)	67 (82%)	
Nonbinary	(1%)	0 (0%)	0 (0%)	1 (2%)	1 (1%)	
URM race/ethnicity						0.39
Yes	(28%)	23 (34%)	16 (30%)	12 (27%)	18 (22%)	
No	(72%)	44 (66%)	37 (70%)	33 (73%)	64 (78%)	
LGBTQI						0.29
Yes	(17%)	7 (10%)	11 (20%)	10 (23%)	13 (16%)	
No	(83%)	60 (90%)	43 (80%)	34 (77%)	69 (84%)	

*Note*: Numbers may not sum to session total due to missing response values.

URM = underrepresented minority; LGBTQI = Lesbian, Gay, Bisexual, Transgender, Queer, Intersex.

* *p*-value for Fisher’s exact test of independence for each participant characteristic across sessions.

### Step 3: Goals and objectives

Each of the four workshop sessions was preceded by a design phase where the DEI facilitator and committee members met bi-weekly to review past session feedback and co-develop curriculum content (slides, facilitator guides, and handouts), plan implementation (advertising, engaging leadership), and address logistical details regarding participants’ needs (venue, time, and food). Feedback, observations, and recommendations gathered from the previous session’s survey results were considered to inform curriculum content.

#### Workshop content

Session 1 aimed to establish a foundation for the workshop series by generating enthusiasm and buy-in, reviewing needs assessment data, and amplifying the departmental commitment to diversity. To facilitate content delivery to a large audience, we held the session immediately after an all-departmental meeting. The first session focused on foundational DEI concepts, unconscious bias, and cultural humility with large and small group activities. Learning objectives included: (a) understand departmental knowledge and perceived value of DEI; (b) communicate departmental diversity goals and initiatives and garner support; (c) introduce foundational diversity concepts; and (d) apply foundational concepts and frameworks to the situational and cultural context of an academic research department.

Session 2 was an extended workshop focused on microaggressions, remarks or behaviors that express hostile, derogatory, and invalidating messages based on race, gender, sexual orientation, and other diverse social identities [33]. It included a didactic presentation on microaggressions and allyship, a worksheet guided self-reflection on microaggression experiences, and anonymized case scenarios of department members’ microaggression experiences presented in guided small group activities. The facilitator emphasized that microaggressions are determined by those who experience harm and stressed the importance of impact vs. intent. The responsibility of the person observing the microaggression to intervene or resolve the situation, rather than the person experiencing the microaggression, was emphasized as part of allyship. Learning objectives included (a) demonstrate the importance of inclusive language by pronouns; (b) understand what microaggressions are and how to spot them; (d) identify and begin applying skills and strategies to engage as an ally.

Session 3 was a hands-on workshop building upon session two that emphasized skill-building to address workplace microaggressions. A didactic presentation on allyship focused on moving from “bystander to upstander” was provided, followed by several small group role-playing activities to practice applying skills [34]. Materials and activities focused on the misconception that the person who is the target of a microaggression is responsible for addressing the harm or giving feedback to others and, instead, stressed the role that bystanders can play as allies to disrupt microaggressions and/or give feedback to those responsible for the harm. Small group activities utilized session 2 case scenarios and tools and materials for disrupting microaggressions [32]. Learning objectives included: (a) demonstrate foundational knowledge of diversity concepts: allyship, power, privilege, and microaggressions; (b) expand awareness about the impact of microaggressions; and (c) practice skills and intervention strategies as an “upstander” to address microaggressions.

Session 4 was rescheduled from a in person session to a virtual session in June 2020 due to the COVID-19 pandemic. Content was modified to address the nation’s racial justice reckoning after George Floyd’s murder and the pandemic’s impact on perpetuating racial disparities. Past foundational work provided an opportunity to come together during this powerful time, process the events, and apply knowledge and skills gained across sessions 1-3. The session was titled “Exploring power, privilege, and race in the COVID-19 era: Opportunities for Allyship.” It began with a reflective exercise, where participants could anonymously share how they were affected by recent events. After a brief didactic review, concepts of power, privilege, and allyship were applied to the co-concurrent topics of anti-black racism and the COVID-19 pandemic. Trained moderators used discussion prompts to facilitate small group discussions via virtual break-out rooms. Participants were pre-assigned to small groups based on their self-identified racial and ethnic identity, with a separate group for department leaders to allow for more candid discussion. Learning objectives included (a) review foundational diversity concepts: power, privilege, and allyship; (b) apply foundational diversity concepts amid COVID-19, shelter-in-place, and events of racial violence; and (c) identify strategies for allyship.

### Step 4: Educational strategies

Workshop content was designed to include a mix of didactic, large-group, and small-group activities to meet the specific session objectives. The DEI facilitator (LHW) provided content expertise. LHW leads diversity education initiatives, serving as a facilitator, keynote speaker, and mentor for emerging diversity champions.

The curriculum content was developed in partnership with the departmental DEI committee members to ensure that content applied to the academic research work environment. Participants received handouts outlining each session’s specific content with space for self-reflections and notes for future reference. Additional materials included: definitions of key terms, resources for reporting discrimination and harassment, and resources for additional DEI learning.

### Step 5: Implementation

#### Workshop administration

To meet our goal of increasing DEI topic knowledge and shifting departmental culture, we sought to engage many departmental members at each workshop session. Participation was voluntary and open to affiliated students, staff, and faculty. To encourage attendance, we advertised the sessions frequently by email, flyers, and announcements at departmental meetings, enlisted support from department leaders, and sent individual invitations to influential members across research groups, projects, and social circles. In-person workshops prior to the pandemic offered breakfast. Lastly, session times were selected to complement core departmental activity and class time to avoid conflicts. Division heads and the Department Chair attended all sessions. Session 1 was held in-person directly following an all-department meeting. To increase accessibility for department members working in different locations, sessions 2–3 were conducted in person with an option for remote participation. Session 4 was conducted solely via Zoom due to local shelter-in-place edicts. Sessions 2–4 didactic portions were recorded for future use.

### Step 6: Evaluation

We administered a pre-session survey and a post-session survey at each workshop session measuring: participant characteristics (i.e., workplace position, supervisor status, time in department, gender identity, URM membership, LGBTQIA + identity, current DEI knowledge and skill level, perception of departmental disparities, and willingness/ability to address and respond to DEI in the workplace). Post-surveys also included open-ended questions to solicit feedback about what worked well, what we could do better and suggestions for DEI topics and experiences for future sessions. Post-survey feedback was reviewed after each session, allowing for some modifications to subsequent session facilitation (e.g., participant requests for additional time in small groups or further clarification on DEI terminology). Surveys were anonymous; personal identifiers were not collected.

### Data analyses

Attendees varied across the four workshop sessions. Furthermore, given that all survey data—both pre- and post-session and across sessions—were anonymous, linking responses to specific individuals was not possible. Consequently, we conducted all analyses using unpaired methods.

We evaluated differences in participant characteristics across sessions using Fisher’s exact test of independence. To evaluate the impact of each session, we calculated summary statistics for all learning evaluation measures pre- and post-session and compared these statistics within a session. For binary measures, we calculated counts and percentages, assessing differences using Fisher’s exact test. For ordinal Likert scale measures, we first determined the distribution of responses. When responses followed a normal distribution, we calculated means and standard deviations and assessed differences between pre- and post-session responses using unpaired *t*-tests. In cases where Likert scale responses were not normally distributed, we calculated medians and interquartile ranges and assessed within-session differences using Wilcoxon rank-sum tests. To evaluate the impact of the training series across the four sessions, we analyzed both binary and ordinal survey responses in two ways. Initially, Fisher’s exact test was applied to assess any differences in the distribution of post-session responses across sessions. Then, trend analyses were conducted using the chi-square trend test for binary responses and the Jonckheere-Terpstra trend test—a non-parametric, rank-based approach—for ordinal responses.

All quantitative analyses were conducted using Stata 16 statistical software [35]. Two authors analyzed the open-ended qualitative data to evaluate other workshop benefits. Results were summarized using reflexive thematic analysis [36]. First, results were coded to identify session successes, failures, and recommended future topics. Next, codes were reviewed to generate themes using an inductive, iterative approach. After reviewing organized data, two authors (AR and LHW) identified and discussed themes with a third author (MM) until reaching agreement regarding workshop benefits. Our study received exempt status from the University of California, San Francisco’s Institutional Review Board.

## Results

Workshop attendance varied by session; range 45 (session 3) to 82 (session 4) (Table 1). Across all sessions, most attendees were staff (64%, including non-faculty academics), and only 25% were faculty, a notable contrast to the department’s overall composition of 41% staff and 51% faculty. Most attendees identified as female (74%) and 28% identified as a member of an URM group, reflecting the composition of the department; 17% identified as LGBTQI+. Characteristics of the workshop attendees did not significantly differ by session (Table 1).

### Knowledge and perceptions of DEI

We compared post-session to presession responses (i.e., within-session differences). We observed statistically significant increases in perceived knowledge about DEI in each of the four sessions (Table 2). Significant pre-post session improvement was observed in having “the skills to improve DEI” in the workplace in sessions 2–4. No statistically significant differences were noted in pre-post session responses for the other ability/willingness to respond domains (i.e., agreement they are responsible for improving DEI or that they plan to work to improve DEI in their workplace).

**Table 2.** Knowledge and perceptions of current diversity, equity and inclusion, pre vs. post for each session, department of epidemiology and biostatistics diversity, equity and inclusion workshops, 2019-2020

	Session 1			Session 2			Session 3			Session 4		
	Pre	Post	*p*-value	Pre	Post	*p*-value	Pre	Post	*p*-value	Pre	Post	*p*-value
											
	*N* = 67	*N* = 60		*N* = 54	*N* = 73		*N* = 45	*N* = 46		*N* = 82	*N* = 80	
Current level of knowledge about DEI, median (IRQ)^*^	3.0 (2.0–3.0)	3.0 (3.0–3.0)	**0.007**	3.0 (2.0–3.0)	3.0 (3.0–3.0)	**0.002**	3.0 (3.0–3.0)	3.0 (3.0–4.0)	**0.039**	3.0 (2.0–3.0)	3.0 (3.0–3.0)	**0.003**
**Yes to? or Perceived disparities in the workplace, Yes?, *n* (%)**												
Recruitment, admissions and hiring practices	18 (27%)	24 (40%)	0.13	24 (44%)	25 (46%)	1.00	16 (36%)	20 (43%)	0.52	41 (50%)	40 (50%)	1.00
Mentoring to support mentees from diverse backgrounds	16 (24%)	20 (33%)	0.32	21 (39%)	32 (59%)	0.054	18 (40%)	18 (39%)	1.00	33 (40%)	36 (45%)	0.63
Progression and promotion; salary	16 (24%)	13 (22%)	0.83	26 (48%)	27 (50%)	1.00	17 (38%)	17 (37%)	1.00	33 (40%)	34 (43%)	0.87
Resource/support allocation	6 (9%)	11 (18%)	0.19	14 (26%)	21 (39%)	0.22	13 (29%)	13 (28%)	1.00	24 (29%)	25 (31%)	0.86
Opportunities for leadership positions	15 (22%)	18 (30%)	0.42	23 (43%)	23 (43%)	1.00	24 (53%)	19 (41%)	0.30	42 (51%)	40 (50%)	1.00
Opportunities to give scientific lectures, workshops	5 (7%)	5 (8%)	1.00	4 (7%)	12 (22%)	0.055	9 (20%)	6 (13%)	0.41	12 (15%)	13 (16%)	0.83
Opportunities to teach courses for our trainees	6 (9%)	2 (3%)	0.28	3 (6%)	8 (15%)	0.20	9 (20%)	7 (15%)	0.59	14 (17%)	11 (14%)	0.66
Opportunities to participate on committees/work groups	3 (4%)	5 (8%)	0.47	7 (13%)	9 (17%)	0.79	7 (16%)	6 (13%)	0.77	10 (12%)	15 (19%)	0.28
Opportunities to participate in lectures, workshops	6 (9%)	5 (8%)	1.00	2 (4%)	8 (15%)	0.093	8 (18%)	5 (11%)	0.38	11 (13%)	10 (13%)	1.00
Degree of agreement with DEI Statements^**^												
Lack of DEI is a problem within my workplace at University of California, San Francisco, mean (SD)	2.6 (0.6)	2.8 (0.5)	0.091	2.6 (0.7)	2.7 (0.8)	0.51	2.7 (0.8)	2.7 (0.7)	0.94	2.8 (0.8)	2.9 (0.9)	0.81
I am comfortable addressing DEI in my workplace, median (IQR)	3.0 (3.0–3.0)	3.0 (3.0–3.0)	0.75	3.0 (3.0–3.5)	3.0 (3.0–4.0)	0.62	3.0 (3.0–3.0)	3.0 (3.0–3.0)	0.58	3.0 (3.0–3.0)	3.0 (3.0–3.0)	0.34
I have the skills to improve DEI in my workplace, mean (SD)	2.8 (0.6)	2.9 (0.6)	0.19	2.7 (0.6)	3.0 (0.5)	**0.011**	2.8 (0.6)	3.1 (0.5)	**0.023**	2.8 (0.5)	3.0 (0.5)	**0.004**
Degree of agreement with DEI Statements^**^												
I am responsible for improving DEI within my workplace, median (IQR)	3.0 (3.0–4.0)	3.5 (3.0–4.0)	0.10	3.0 (3.0–4.0)	4.0 (3.0–4.0)	0.27	3.0 (3.0–4.0)	4.0 (3.0–4.0)	0.78	4.0 (3.0–4.0)	3.0 (3.0–4.0)	0.50
I plan to work to improve DEI in my workplace, median, IQR	3.0 (3.0–4.0)	4.0 (3.0–4.0)	0.16	4.0 (3.0–4.0)	4.0 (3.0–4.0)	0.78	3.0 (3.0–4.0)	4.0 (3.0–4.0)	0.25	3.0 (3.0–4.0)	3.0 (3.0–4.0)	0.76

*p*-values for comparisons of binary variables (i.e. perceived disparities in the workplace) between pre and post based on Fisher’s exact test; *p*-values for comparisons of continuous variables (i.e. Likert scales) between pre and post based on two sample *t*-tests if normally distributed responses and Wilcoxon rank-sum tests if non-normally distributed; (paired tests not used because individual-level identifiers were not collected).

IQR = interquartile range; SD = standard deviation; DEI = diversity, equity, and inclusion.

* 4-point Likert scale ranging from 1 “Not knowledgeable at all” to 4 “Very knowledgeable.”

** 4-point Likert scale ranging from 1 “Strongly disagree” to 4 “Strongly agree.”

We also assessed differences in post-session responses across the four sessions (i.e., between-session differences) and found a statistically significant difference for perceived disparities in mentoring that supports mentees from diverse backgrounds (*p* = 0.041), perceived disparities in progression and promotion (salary) (*p* = 0.011), and perception that the lack of DEI is a problem within the workplace (*p* = 0.034). A linear trend across sessions was observed for perceived disparities in opportunities for leadership positions (*p* = 0.026), indicating a growing perception of disparities in this area (Table 3).

**Table 3.** Post-workshop knowledge and perceptions of diversity, equity and inclusion topics, overall and by sessions, [UNIVERSITY] diversity, equity, and inclusion workshops, 2019–2020

		Session 1	Session 2	Session 3	Session 4		
	Overall	*N* = 60	*N* = 54	*N* = 46	*N* = 80	*p*-value, Fisher’s exact test*	*p*-value, test for trend**
Current level of knowledge about DEI, Likert scale						0.32	0.48
A little knowledgeable	(8%)	8 (13%)	4 (7%)	2 (4%)	6 (8%)		
Somewhat knowledgeable	(73%)	44 (73%)	38 (70%)	31 (67%)	62 (78%)		
Very knowledgeable	(19%)	8 (13%)	12 (22%)	13 (28%)	12 (15%)		
**Perceived disparities in department, n(%) affirming**							
Recruitment, admissions and hiring practices	(45%)	24 (40%)	25 (46%)	20 (43%)	40 (50%)	0.69	0.29
Mentoring to support mentees from diverse backgrounds	(44%)	20 (33%)	32 (59%)	18 (39%)	36 (45%)	**0.041**	0.55
Progression and promotion; salary	(38%)	13 (22%)	27 (50%)	17 (37%)	34 (43%)	**0.011**	0.062
Resource/support allocation	(29%)	11 (18%)	21 (39%)	13 (28%)	25 (31%)	0.10	0.26
Opportunities for leadership positions	(42%)	18 (30%)	23 (43%)	19 (41%)	40 (50%)	0.13	**0.026**
Opportunities to give scientific lectures, workshops	(15%)	5 (8%)	12 (22%)	6 (13%)	13 (16%)	0.21	0.44
Opportunities to teach courses for our trainees	(12%)	2 (3%)	8 (15%)	7 (15%)	11 (14%)	0.092	0.092
Opportunities to participate on committees/work groups	(15%)	5 (8%)	9 (17%)	6 (13%)	15 (19%)	0.34	0.13
Opportunities to participate in lectures, workshops	(12%)	5 (8%)	8 (15%)	5 (11%)	10 (13%)	0.76	0.62
**Degree of agreement with DEI Statements, Likert scale**							
Lack of DEI is a problem within my workplace at University of California, San Francisco						**0.034**	0.57
Strongly disagree	(4%)	0 (0%)	3 (6%)	1 (2%)	5 (7%)		
Disagree	(28%)	13 (25%)	16 (31%)	14 (33%)	20 (27%)		
Agree	(52%)	36 (69%)	25 (48%)	23 (53%)	31 (41%)		
Strongly agree	(16%)	3 (6%)	8 (15%)	5 (12%)	19 (25%)		
I am responsible for improving DEI within my workplace						0.96	0.73
Disagree	(5%)	3 (5%)	2 (4%)	3 (7%)	3 (4%)		
Agree	(44%)	26 (45%)	22 (41%)	19 (42%)	37 (48%)		
Strongly agree	(51%)	29 (50%)	30 (56%)	23 (51%)	37 (48%)		
I am comfortable addressing DEI in my workplace						0.34	0.92
Disagree	(14%)	7 (12%)	11 (21%)	5 (11%)	9 (11%)		
Agree	(66%)	40 (70%)	27 (51%)	31 (67%)	56 (71%)		
Strongly agree	(21%)	10 (18%)	15 (28%)	10 (22%)	14 (18%)		
I have the skills to improve DEI in my workplace						0.28	0.23
Disagree	(14%)	13 (23%)	6 (11%)	4 (9%)	9 (12%)		
Agree	(73%)	37 (65%)	41 (77%)	32 (70%)	59 (77%)		
Strongly agree	(14%)	7 (12%)	6 (11%)	10 (22%)	9 (12%)		
I plan to work to improve DEI in my workplace						0.57	0.32
Disagree	(0%)	1 (2%)	0 (0%)	0 (0%)	0 (0%)		
Agree	(48%)	25 (44%)	24 (45%)	20 (44%)	43 (54%)		
Strongly agree	(52%)	31 (54%)	29 (55%)	25 (56%)	36 (46%)		

DEI = diversity, inclusion, and equity.

* *p*-values based on Fisher’s exact test for any difference in the response distribution between sessions.

** *p*-values based on test for trend across the four sessions (chi-square test for trend for dichotomous responses; Jonckheere–Terpstra non-parametric, rank-based test for trend for ordinal responses, with exact p-value from Monte–Carlo permutations).

Finally, we investigated whether the observed differences in perceived disparities across sessions, namely mentoring, progression and promotion, and opportunities for leadership positions, varied depending on identification with an URM group. We found that the observed significant differences across sessions were among the URM participant groups, but not among non-URM groups (Supplemental Table 2).

### Open-ended feedback

Themes for what worked well were consistent across sessions. Having an excellent facilitator was the most common theme, particularly in the first two sessions. Respondents praised the facilitator as an engaging, dynamic, and funny; one attendee said she made “a difficult topic easier to handle.” Other common positive themes included small group discussions and good real-life examples, both of which increased across sessions. Themes for what could have improved varied across sessions. The most common themes for improvement were the need for more tangible skills (session 1), more time and better time management for discussions (sessions 2 and 3), and the need for more sessions (session 4). Desire for more small-group interaction time was noted across all sessions. The most requested topic for future sessions was how to personally improve/promote DEI in the workplace (session 1) and microaggressions, responding to conflicts, and allyship (sessions 2–4).

## Discussion

We designed and implemented a successful departmental DEI workshop series at a large academic institution. Our goals were to increase the overall understanding of core DEI concepts and increase skills to improve DEI. The series was one step in our larger departmental approach to changing departmental culture and deconstructing academic systems for enhanced inclusion and diversity.

Our evaluation identified several positive findings indicating the workshops’ success. There was an overarching increase in the workshops’ effectiveness in promoting DEI knowledge and skill development. A more in-depth analysis revealed that perceptions of DEI disparities in the workplace were only significant for underrepresented participants. Finally, open text feedback affirmed that the workshop design aligned with researched best practices for comprehensive DEI initiatives and approaches [16]. In addition, we identified ways to improve future DEI efforts in our department which may inform DEI curriculum development and implementation at similar institutions.

The finding that the DEI workshops had positive effects on knowledge and skill development was consistent with existing literature on the efficacy of diversity training, especially literature that lauds the positive effect that diversity training has on cognitive learning [16]. This also suggests a benefit to attending one or multiple workshops. Future diversity training may model a curriculum series of DEI topics to build upon learning and provide more opportunities to practice skills and demonstrate sustained commitment to DEI initiatives. Additionally, while attendance at all DEI workshops in a curricula series should be encouraged, participants will benefit from attending one or more workshops.

Analyses assessing perceived disparities in the workplace indicated significant increases across sessions; however, further analysis revealed that these findings were only significant for URM participants. Over the course of the DEI workshops, URM participants reported perceived disparities in mentoring for mentees from diverse backgrounds, perceived disparities in professional career progression and promotion, and confirmed the perception that the lack of DEI is a problem within the workplace. A possible understanding can be drawn from the theory of stereotype threat [37]. Participating in the DEI workshop series may have unearthed the previously internalized experiences of URM attendees (e.g., “I can’t say for sure that I am being treated differently from my non-URM peers, but I feel overlooked for professional opportunities”), thus transforming perceived disparities from an individual challenge to a collective experience of inequity (e.g., “While it is still disheartening to hear that other URM junior faculty and researchers struggle with mentorship, it’s helpful to know that I’m not the only one”) [38].

Open text feedback also illuminated workshop strengths on the efficacy of comprehensive diversity training that were consistent with the literature, including themes such as facilitation by experts, content tailored to the organization, inclusion of employees from all levels, support from senior leadership, and connection to the group’s operating goals [17]. In addition, the department had a previously established diversity committee made up of both faculty and staff members who independently participated in campus professional development for DEI foundations. The department chair supported the committee and attended every workshop in the DEI training series. Together, these factors likely contributed to the overall success of the DEI training series.

There were limitations to our study. First, our data do not allow us to assess the workshop’s impact on behaviors or whether participants practiced skills introduced in the workshop. Also, we could not discern unique attendees from the total and how many attended all four sessions. Changes in behavior can take longer to observe which may explain why we did not see significant improvements in attendees’ agreement that they are responsible for improving DEI within their workplace or that they plan to work to improve DEI in their workplace. Discussions on challenges transferring learning to behavior often cite the need for longitudinal initiatives, leadership accountability, and strategies to support new behaviors [39]. Our department is conducting a 1-year post-series questionnaire to evaluate longer-term changes in knowledge and skills and whether participants have applied DEI skills. On a broad institutional level, the academic university implements many of the best practices listed in Enders and colleagues’ recommendations for a comprehensive DEI action plan, such as a biannual climate survey and incorporating best practices in addressing bias in search committees. However, because we operate in a very large matrixed health science institution, it is imperative that DEI action plans are further refined at the departmental level to address unique needs and maximize impact [40].

Overall, curating a multipart DEI workshop series successfully improved participants’ knowledge and attitudes and aligned with core values of culturally congruent training for public health practitioners and researchers. As professionals, we educate the next generation of leaders and engage in service, research, and public policy that frequently shape narratives surrounding historically underrepresented and vulnerable communities. Additionally, as structural racism influences health outcomes but also the scholarly and academic knowledge about populations, etiology, and health disparities, diversity education and equity, initiatives must become centered in these endeavors. DEI initiatives are a collective responsibility, but often rely on the energy provided by historically excluded groups. While our attendees were largely representative of our department demographics, future efforts should be mindful of the need to increase engagement of faculty, especially from male and/or White identified faculty members. While our curricula was developed to engage a broad audience of academic researchers, one insight learned is the importance of balancing these collaborative sessions with additional affinity-style spaces and curricula to address the needs for various subgroups (e.g., staff, faculty, trainees, URM, women, LGBTQI +, etc.)

## Supporting information

Hill Weller et al. supplementary material Hill Weller et al. supplementary material

## Supplementary material

To view supplementary material for this article, please visit https://doi.org/10.1017/cts.2024.6

## References

[1] DV Hunt , Yee L , Prince S , Dixon-Fyle S. Delivering growth through diversity in the workplace|McKinsey. 2018. https://www.mckinsey.com/capabilities/people-and-organizational-performance/our-insights/delivering-through-diversity. Accessed October 19, 2023.

[2] Rock D , Grant H. Why diverse teams are smarter. Harv Bus Rev. 2016;4. https://hbr.org/2016/11/why-diverse-teams-are-smarter. Accessed August 2021.

[3] Gomez LE , Bernet P. Diversity improves performance and outcomes. J Natl Med Assoc. 2019;111:383–392. doi: 10.1016/j.jnma.2019.01.006. 30765101

[4] Borrell LN , Crawford ND. Racial and ethnic inequities in health: examining the contributions of the American Journal of Epidemiology to Advancing the Science. Am J Epidemiol. 2023;192:1827–1834. doi: 10.1093/aje/kwac069. 35380604

[5] Fortuna LR , Tolou-Shams M , Robles-Ramamurthy B , Porche MV. Inequity and the disproportionate impact of COVID-19 on communities of color in the United States: the need for a trauma-informed social justice response. Psychol Trauma Theory Res Pract Policy. 2020;12:443–445. doi: 10.1037/tra0000889. PMC8243721 32478545

[6] Krieger N. Structural racism, police brutality, plutocracy, climate change—and time for health justice, democratic governance, and an equitable, sustainable future. Am J Public Health. 2020;110:1620–1623. doi: 10.2105/AJPH.2020.305886. 32816556 PMC7542259

[7] Nguyen TT , Criss S , Dwivedi P , et al. Exploring U.S. shifts in anti-Asian sentiment with the emergence of COVID-19. Int J Environ Res Public Health. 2020;17:7032. doi: 10.3390/ijerph17197032. 32993005 PMC7579565

[8] Collins FS , Adams AB , Aklin C , et al. Affirming NIH’s commitment to addressing structural racism in the biomedical research enterprise. Cell. 2021;184:3075–3079. doi: 10.1016/j.cell.2021.05.014. 34115967

[9] Castrucci BC. The “10 essential public health services” is the common framework needed to communicate about public health. Am J Public Health. 2021;111:598–599. doi: 10.2105/AJPH.2021.306189. 33689415 PMC7958075

[10] Alang S , Hardeman R , Karbeah J , et al. White supremacy and the core functions of public health. Am J Public Health. 2021;111:815–819. doi: 10.2105/AJPH.2020.306137. 33826395 PMC8033999

[11] The American Association of Medical Colleges. Diversity in medicine: facts and figures 2019. https://www.aamc.org/data-reports/workforce/report/diversity-medicine-facts-and-figures-2019. Accessed August 24, 2021.

[12] Sellers K , Leider JP , Gould E , et al. The State of the US Governmental Public Health Workforce, 2014–2017. Am J Public Health. 2019;109:674–680. doi: 10.2105/AJPH.2019.305011. 30896986 PMC6459653

[13] Harvey M , Neff J , Knight KR , et al. Structural competency and global health education. Glob Public Health. 2022;17:341–362. doi: 10.1080/17441692.2020.1864751. 33351721

[14] Metzl JM , Hansen H. Structural competency: theorizing a new medical engagement with stigma and inequality. Soc Sci Med. 2014;103:126–133. doi: 10.1016/j.socscimed.2013.06.032. 24507917 PMC4269606

[15] Pendry LF , Driscoll DM , Field SCT. Diversity training: putting theory into practice. J Occup Organ Psychol. 2007;80:27–50. doi: 10.1348/096317906X118397.

[16] Bezrukova K , Spell CS , Perry JL , Jehn KA. A meta-analytical integration of over 40 years of research on diversity training evaluation. Psychol Bull. 2016;142:1227–1274. doi: 10.1037/bul0000067. 27618543

[17] Bendick M , Egan ML , Lofhjelm SM. Workforce diversity training: from anti-discrimination compliance to organizational development. Hum Resour Plan. 2001;24:10–25.

[18] Shandera S , Matsick JL , Hunter DR , Leblond L. RASE: modeling cumulative disadvantage due to marginalized group status in academia. PloS One. 2021;16:e0260567. doi: 10.1371/journal.pone.0260567. 34914741 PMC8675700

[19] James L , Venable T , Caro A , et al. Development of a clinical and translational research curriculum for undergraduate students. J Clin Transl Sci. 2023;7:e118. doi: 10.1017/cts.2023.532. 37313383 PMC10260337

[20] Enders FT , Golembiewski EH , Orellana M , Silvano CJ , Sloan J , Balls-Berry J. The hidden curriculum in health care academia: an exploratory study for the development of an action plan for the inclusion of diverse trainees. J Clin Transl Sci. 2021;5:e203. doi: 10.1017/cts.2021.867. 35047215 PMC8727720

[21] Balls-Berry JE , Rubio D , Aguilar-Gaxiola S , et al. 124 the formation of the ACTS diversity, equity, and inclusion committee to increase belonging. J Clin Transl Sci. 2023;7:37. doi: 10.1017/cts.2023.206.

[22] Enders F , Wurth R , Orellana M , Menser T. 194 introducing the new justice, equity, diversity, and inclusion special interest group. J Clin Transl Sci. 2023;7:60. doi: 10.1017/cts.2023.270.

[23] Rubio D , Norman MK , Mayowski CA , et al. Leading emerging and diverse scientists to success: results from LEADS alumni. J Clin Transl Sci. 2023;7:e39. doi: 10.1017/cts.2022.513. 36845299 PMC9947607

[24] Strekalova YAL , Qin YS , Sharma S , et al. The black voices in research curriculum to promote diversity and inclusive excellence in biomedical research. J Clin Transl Sci. 2021;5:e206. doi: 10.1017/cts.2021.869. 35047217 PMC8727711

[25] Enders FT , Golembiewski EH , Pacheco-Spann LM , Allyse M , Mielke MM , Balls-Berry JE. Building a framework for inclusion in health services research: development of and pre-implementation faculty and staff attitudes toward the diversity, equity, and inclusion (DEI) plan at mayo clinic. J Clin Transl Sci. 2021;5:e88. doi: 10.1017/cts.2020.575. 34007470 PMC8111694

[26] Ford CL , Griffith DM , Bruce MA , Gilbert KL. Racism: Science & Tools for the Public Health Professional. Washington, D.C.: American Public Health Association, 2019.

[27] Ford CL , Airhihenbuwa CO. Critical race theory, race equity, and public health: toward antiracism praxis. Am J Public Health. 2010;100:S30–S35. doi: 10.2105/AJPH.2009.171058. 20147679 PMC2837428

[28] Van Schalkwyk SC , Hafler J , Brewer TF , et al. Transformative learning as pedagogy for the health professions: a scoping review. Med Educ. 2019;53:547–558. doi: 10.1111/medu.13804. 30761602

[29] Kaufman DM , Mann KV. Teaching and learning in medical education: how theory can inform practice. In: Swanwick T , ed. Understanding Medical Education: Evidence, Theory and Practice. Hoboken, New Jersey: Wiley-Blackwell, 2010:16–36.

[30] Thomas PA , Kern DE , Hughes MT , Chen BY. Curriculum Development for Medical Education: A Six Step Approach. Maryland, US: Johns Hopkins University Press, 2015.

[31] Gwayi-Chore MC , Del Villar EL , Fraire LC , et al. “Being a person of color in this institution is exhausting”: Defining and optimizing the learning climate to support diversity, equity, and inclusion at the university of Washington school of public health. Front Public Health. 2021;9:642477. doi: 10.3389/fpubh.2021.642477. 33937172 PMC8082071

[32] Harrison-Bernard LM , Augustus-Wallace AC , Souza-Smith FM , Tsien F , Casey GP , Gunaldo TP. Knowledge gains in a professional development workshop on diversity, equity, inclusion, and implicit bias in academia. Adv Physiol Educ. 2020;44:286–294. doi: 10.1152/advan.00164.2019. 32484403 PMC7642839

[33] Ehie O , Muse I , Hill L , Bastien A. Professionalism: microaggression in the healthcare setting. Curr Opin Anaesthesiol. 2021;34:131–136. doi: 10.1097/ACO.0000000000000966. 33630771 PMC7984763

[34] Ho CP , Chong A , Narayan A , et al. Mitigating Asian American bias and xenophobia in response to the coronavirus pandemic: how you can be an upstander. J Am Coll Radiol. 2020;17:1692–1694. doi: 10.1016/j.jacr.2020.09.030. 33035504 PMC7538066

[35] StataCorp. Stata Statistical Software: Release 16. College Station, TX: StataCorp LLC, 2019.

[36] Braun V , Clarke V. Using thematic analysis in psychology. Qual Res Psychol. 2006;3:77–101. doi: 10.1191/1478088706qp063oa.

[37] Casad BJ , Bryant WJ. Addressing stereotype threat is critical to diversity and inclusion in organizational psychology. Front Psychol. 2016;7:8. doi: 10.3389/fpsyg.2016.00008. 26834681 PMC4718987

[38] Ajayi AA , Rodriguez F , de Jesus Perez V. Prioritizing equity and diversity in academic medicine faculty recruitment and retention. JAMA Health Forum. 2021;2:e212426. doi: 10.1001/jamahealthforum.2021.2426. 36218655 PMC9552628

[39] Kirkpatrick DL , Kirkpatrick JD. Evaluating Training Programs: The Four Levels. 3rd ed. San Francisco, CA: Berrett-Koehler Publishers, 2006.

[40] Enders FT , Golembiewski EH , Orellana MA , et al. Changing the face of academic medicine: an equity action plan for institutions. J Clin Transl Sci. 2022;6:e78. doi: 10.1017/cts.2022.408. 35874036 PMC9280456

