# Supplementary material for: Lessons learned from implementing a diversity, equity, and inclusion curriculum for health research professionals at a large academic research institution
Source: J Clin Transl Sci. 2024 Jan 12;8(1):e22. doi: 10.1017/cts.2024.6 (PMC10879992; doi:10.1017/cts.2024.6)
Supplement: Hill Weller et al. supplementary material [file S2059866124000062sup001.docx]

Supplemental Materials

Supplemental Table 1: [UNIVERSITY] Department of Epidemiology and Biostatistics (DEB) Diversity, Equity, Inclusion Needs Assessment Survey Results (Electronic survey prior to workshops to inform workshop content), August 2019

|  | Total |
| --- | --- |
|  | N=100 |
| Current appointment in the [UNIVERSITY] DEB |  |
| Staff or Non-Faculty Academic | 50 (50%) |
| Faculty | 34 (34%) |
| Trainee (PhD Student or Post Doc) | 16 (16%) |
| Years in the [UNIVERSITY] DEB** |  |
| Less than 5 years | 71 (71%) |
| 5 or more years | 28 (28%) |
| DEI training in past 5 years** |  |
| Yes | 34 (34%) |
| No | 62 (62%) |
| Type of DEI training in past 5 years. (Mark all that apply.) |  |
| [UNIVERSITY] Diversity, Equity and Inclusion Champion Training | 10 (10%) |
| [UNIVERSITY] Diversity & Inclusion Staff Certificate Program | 3 ( 3%) |
| DEB-hosted training | 11 (11%) |
| Introduction to/foundations of diversity, equity and inclusion | 9 ( 9%) |
| Increasing diversity in the workplace | 6 ( 6%) |
| Unconscious bias training | 13 (13%) |
| LGBTQIA+ | 1 ( 1%) |
| Sexual harassment & sexual violence prevention | 15 (15%) |
| Discrimination in the workplace | 7 ( 7%) |
| Cultural humility and cultural competence | 7 ( 7%) |
| Diversity & Inclusion Foundations | 2 ( 2%) |
| Allyship training | 2 ( 2%) |
| Inclusive leadership | 1 ( 1%) |
| Recognizing/addressing microaggressions in the workplace | 4 ( 4%) |
| I can’t recall the specific type of training | 7 ( 7%) |
| Why did you take these training(s)? (Mark all that apply.) |  |
| Personal interest | 22 (22%) |
| Requirement for current position | 15 (15%) |
| Professional development | 16 (16%) |
| Satisfaction with [UNIVERSITY] DEI training (scale 1-5)*, median (IQR) | 4 (3-5) |
| Priority rating for areas you would like to learn more about**: |  |
| Understanding why diversity is important in the workplace |  |
| Low | 37 (37%) |
| Medium | 33 (33%) |
| High | 27 (27%) |
| Increasing diversity in the workplace/recruitment strategies |  |
| Low | 11 (11%) |
| Medium | 32 (32%) |
| High | 56 (56%) |
| Dimensions of diversity, from sexual/gender to socioeconomic |  |
| Low | 19 (19%) |
| Medium | 47 (47%) |
| High | 31 (31%) |
| How to mentor individuals from diverse backgrounds |  |
| Low | 10 (10%) |
| Medium | 30 (30%) |
| High | 57 (57%) |
| Promotion/progression of faculty/staff from diverse backgrounds |  |
| Low | 11 (11%) |
| Medium | 42 (42%) |
| High | 44 (44%) |
| Sexual harassment and sexual violence prevention |  |
| Low | 31 (31%) |
| Medium | 36 (36%) |
| High | 29 (29%) |
| Recognizing and actively addressing “hidden bias” in the workplace (Unconscious bias) |  |
| Low | 3 ( 3%) |
| Medium | 35 (35%) |
| High | 60 (60%) |
| Building skills for learning and understanding cultural identities and differences (Cultural humility and cultural competence) |  |
| Low | 10 (10%) |
| Medium | 41 (41%) |
| High | 48 (48%) |
| How to support diverse colleagues in the workplace (Allyship training) |  |
| Low | 8 ( 8%) |
| Medium | 35 (35%) |
| High | 53 (53%) |
| Developing skills to create inclusive climates that motivate and engage teams (Inclusive leadership) |  |
| Low | 5 ( 5%) |
| Medium | 35 (35%) |
| High | 57 (57%) |
| Recognizing and avoiding subtle phrases and actions that can damage work culture (microaggressions in the workplace) |  |
| Low | 8 ( 8%) |
| Medium | 38 (38%) |
| High | 50 (50%) |

* 5-point Likert scale ranging from 1 “Not at all Satisfied” to 5 “Extremely Satisfied”

** Numbers do not sum to session total due to missing response values.

**Supplemental Table 2. Post-hoc evaluation of observed increases in perceived disparities, stratified by underrepresented racial or ethnic minority group**

|  |  |  |  |  |  |  |
| --- | --- | --- | --- | --- | --- | --- |
|  | **Session 1** | **Session 2** | **Session 3** | **Session 4** | **p-value, Fisher's exact test*** | **p-value, test for trend**** |
| **Perceived disparities in DEB, n(%) affirming** |  |  |  |  |  |  |
| Mentoring to support mentees from diverse backgrounds |  |  |  |  |  |  |
| Underrepresented racial or ethnic minority (URM) participants | 9 (41%) | 12 (86%) | 3 (25%) | 12 (55%) | **0.010** | 0.92 |
| Non-URM participants | 11 (29%) | 20 (51%) | 15 (44%) | 24 (41%) | 0.25 | 0.45 |
| Progression and promotion; salary |  |  |  |  |  |  |
| Underrepresented racial or ethnic minority (URM) participants | 2 (9%) | 7 (50%) | 4 (33%) | 11 (50%) | **0.012** | **0.01** |
| Non-URM participants | 11 (29%) | 20 (51%) | 13 (38%) | 23 (40%) | 0.26 | 0.63 |
| Opportunities for leadership positions |  |  |  |  |  |  |
| Underrepresented racial or ethnic minority (URM) participants | 7 (32%) | 5 (36%) | 4 (33%) | 12 (55%) | 0.44 | 0.15 |
| Non-URM participants | 11 (29%) | 18 (46%) | 15 (44%) | 28 (48%) | 0.27 | 0.10 |

*p-values based on Fisher's exact test assessing any difference in the response distribution between sessions

**p-values for test for trend across the sessions (chi-square test for trend for binary responses; Jonckheere-Terpstra non-parametric, rank-based test for trend with exact p-value based on Monte Carlo permutations for ordinal responses)
